# Q-FISH Measurement of Hepatocyte Telomere Lengths in Donor Liver and Graft after Pediatric Living-Donor Liver Transplantation: Donor Age Affects Telomere Length Sustainability

**DOI:** 10.1371/journal.pone.0093749

**Published:** 2014-04-11

**Authors:** Youichi Kawano, Naoshi Ishikawa, Junko Aida, Yukihiro Sanada, Naotaka Izumiyama-Shimomura, Ken-ichi Nakamura, Steven S. S. Poon, Koshi Matsumoto, Koichi Mizuta, Eiji Uchida, Takashi Tajiri, Hideo Kawarasaki, Kaiyo Takubo

**Affiliations:** 1 Department of Surgery, Nippon Medical School, Tokyo, Japan; 2 Research Team for Geriatric Pathology, Tokyo Metropolitan Institute of Gerontology, Tokyo, Japan; 3 Department of Transplant Surgery, Jichi Medical University, Tochigi, Japan; 4 Terry Fox Laboratory, British Columbia Cancer Research Centre, Vancouver, British Columbia, Canada; 5 Department of Clinical Pathology, Ebina General Hospital, Kanagawa, Japan; University of Newcastle, United Kingdom

## Abstract

Along with the increasing need for living-donor liver transplantation (LDLT), the issue of organ shortage has become a serious problem. Therefore, the use of organs from elderly donors has been increasing. While the short-term results of LDLT have greatly improved, problems affecting the long-term outcome of transplant patients remain unsolved. Furthermore, since contradictory data have been reported with regard to the relationship between donor age and LT/LDLT outcome, the question of whether the use of elderly donors influences the long-term outcome of a graft after LT/LDLT remains unsettled. To address whether hepatocyte telomere length reflects the outcome of LDLT, we analyzed the telomere lengths of hepatocytes in informative biopsy samples from 12 paired donors and recipients (grafts) of pediatric LDLT more than 5 years after adult-to-child LDLT because of primary biliary atresia, using quantitative fluorescence *in situ* hybridization (Q-FISH). The telomere lengths in the paired samples showed a robust relationship between the donor and grafted hepatocytes (*r* = 0.765, *p* = 0.0038), demonstrating the feasibility of our Q-FISH method for cell-specific evaluation. While 8 pairs showed no significant difference between the telomere lengths for the donor and the recipient, the other 4 pairs showed significantly shorter telomeres in the recipient than in the donor. Multiple regression analysis revealed that the donors in the latter group were older than those in the former (*p* = 0.001). Despite the small number of subjects, this pilot study indicates that donor age is a crucial factor affecting telomere length sustainability in hepatocytes after pediatric LDLT, and that the telomeres in grafted livers may be elongated somewhat longer when the grafts are immunologically well controlled.

## Introduction

Liver transplantation (LT) has been carried out worldwide for patients with end-stage liver failure [1]. Since the supply of cadaveric liver grafts is far short of the number of patients awaiting transplantation, living-donor liver transplantation (LDLT) has emerged as a critical surgical option for the patients. Along with the increasing need for LDLT, the issue of organ shortage continues to extend worldwide and has become a serious problem. Therefore, the use of organs from marginal donors, such as the elderly, has been increasing [2]. The short-term results of LDLT have greatly improved because of advances in surgical techniques, antiviral therapy and, particularly, immunosuppressive regimens [3], [4], [5], [6]. However, new problems, including recurrence of hepatitis C or hepatocellular carcinoma and development of *de novo* malignancies, have arisen as major problems affecting the long-term outcome of transplant patients [7]. Ensuring that a transplanted organ has a potentially sufficient working life remains one of the most important issues associated with LDLT [8], [9], [10]. In this context, a fundamental question has arisen concerning the senescence (aging process) of organs that are transplanted from elderly donors into young recipients. LDLT provides a unique and valuable means of answering this question, because it allows us to compare the characteristics of hepatocyte samples from an originally identical liver, part of which continues to be viable and functional in the donor, and another part that has functioned in a young recipient for a number of years after transplantation.

Human telomeric DNA is considered to protect chromosomes against degeneration, fusion, and loss [11]. In many human organs and tissues, including the liver [12], [13], *in vivo* telomere shortening occurs with aging, and is accelerated by various environmental factors such as oxidative stress, chronic inflammation, social stress, alcohol, and transplantation [14], [15], [16], [17], [18], [19], [20]. The telomere hypothesis of cellular aging suggests that when telomere shortening reaches a critical level, a DNA damage checkpoint mechanism may be initiated and the cells stop dividing [21], [22]. Therefore, measurement of telomere length is expected to provide information for evaluating the life span of a cell.

Since Lansdorp et al. devised the quantitative fluorescence *in situ* hybridization (Q-FISH) method [23], several studies of telomere length using various Q-FISH methods have been reported by different groups including us [16], [24], [25], [26], [18]. However there have been no reports of telomere length measurement in liver grafts after LDLT. We had an opportunity to study 12 pairs of samples obtained at the same time by needle biopsy from the donors and recipients more than after 5 years after LDLT. To address the question of whether telomere shortening is accelerated in the graft relative to the native liver remaining in the donor after pediatric LDLT, we used Q-FISH to measure the telomere length in hepatocytes.

## Materials and Methods

### Patients

We offered all of the LDLT patients (donors and recipients) being treated at our institution a chance to participate in this pilot study, and twelve pairs volunteered to take part. These twelve cases were considered suitable for our study protocol, which involved examination of a pair of liver biopsy specimens from both the donor and recipient after a follow-up period of more than 5 years after LDLT. No other selection criteria were adopted. These informative cases were obtained from among 134 recipients who had undergone LDLT at the Jichi Medical University Hospital between May 2001 and March 2009. The overall patient survival rate in our department was 95.0% (134/141). The ages, relationships (parent to child in all pairs) and post-LT survivals of the 12 pairs of donors and recipients are listed in Table 1. All of the patients examined in this study had end-stage biliary atresia, a condition that accounted for 73% of total LDLT operations performed at our hospital. Hepatocellular reserve capacity after Kasai portoenterostomy is usually evaluated in terms of the pediatric end-stage liver disease (PELD) score [27], [28]. The PELD score is calculated on the basis of several objective values, the main components being age, growth failure (based on sex, height, and weight), albumin (g/dl), prothrombin time (international normalized ratio), and total bilirubin (mg/dl) (shown in Table S2).

**Table 1:** Characteristics of donors and patients.

Group	Pair No.		Age at LDLT	Relation	Biopsy Duration from LDLT	Blood type	HLA mismatch number	Median value of NTCR	*p* value
Lower NTCR Group		Donor	36y8m	Father		A+		1.51	
	1				12y6m		3		*p*<0.001
		Recipient	1y3m	Daughter		A+		1.07	
		Donor	44y10m	Father		O+		0.42	
	2				11y4m		2		*p*<0.001
		Recipient	8y4m	Son		O+		0.27	
		Donor	39y0m	Father		A+		1.04	
	3				5y6m		3		*p* = 0.015
		Recipient	1y10m	Daughter		A+		0.78	
		Donor	42y3m	Mother		B+		1.13	
	4				5y6m		3		*p*<0.001
		Recipient	9m	Daughter		B+		0.73	
Comparable NTCR Group		Donor	36y7m	Father		O+		1.05	
	5				16y10m		3		*p* = 0.265
		Recipient	9m	Daughter		O+		0.95	
		Donor	35y7m	Father		B+		0.57	
	6				15y3m		3		*p* = 0.948
		Recipient	1y7m	Daughter		AB+		0.53	
		Donor	38y4m	Father		O+		0.99	
	7				5y4m		3		*p* = 0.366
		Recipient	7y3m	Daughter		O+		0.96	
		Donor	37y9m	Stepfather		B+		0.37	
	8				13y0m		6		*p* = 0.065
		Recipient	4y1m	Son		B+		0.46	
		Donor	28y7m	Mother		O+		0.56	
	9				10y6m		2		*p* = 0.109
		Recipient	1y0m	Son		A+		0.77	
		Donor	32y1m	Father		A+		1.03	
	10				8y9m		3		*p* = 0.056
		Recipient	2y6m	Daughter		A+		1.2	
		Donor	34y8m	Father		B+		0.88	
	11				6y7m		3		*p* = 0.115
		Recipient	2y7m	Daughter		B+		1.02	
		Donor	29y5m	Father		A+		0.77	
	12				5y5m		2		*p* = 0.342
		Recipient	1y5m	Daughter		A+		0.82	

NTCR: normalized telomere centromere ratio.

Lower NTCR group: Median NTCRs of the recipient hepatocyte were significantly lower than those of the donor.

Comparable NTCR group: Median NTCRs of the recipient hepatocyte were neither significantly lower nor higher than those of the donor.

Median donor age and median recipient age at the time of LDLT were 36.6 y (28.6 y to 44.8 y) and 1.7 y (0.8 y to 8.3 y), respectively.

Median donor age and median recipient age at the time of biopsy were 46.2 y (34.8 y to 56.2 y) and 12.0 y (6.3 y to 19.7 y), respectively.

The median interval from LDLT to biopsy was 9.6 y.

The ethics committees of Jichi Medical University and Tokyo Metropolitan Institute of Gerontology approved the use of liver biopsy for this study. The participants provided written informed consent before each liver biopsy in accordance with the Declaration of Helsinki. Parental written permission was obtained when the patients were younger than 20 years old.

### Surgical procedure and postoperative management

The surgical procedure (left side liver graft) and postoperative management (immunosuppression started using tacrolimus and steroid) for LDLT were basically the same as described previously [29]. The immunosuppression status of the recipients at the time of biopsy is summarized in Table 2, and the most recent status is summarized in Table S3. All of the grafted livers from donors were confirmed to be histologically normal by biopsy at the time of LDLT.

**Table 2:** Clinical and pathological status of donors and recipients.

Group	Pair No.		Major complication of recipient	Pathological findings	Immunosuppression status
Lower NTCR Group		Donor		(-)	
	1	Recipient	Chronic hepatitis	Mild interface hepatitis	Tacrolimus: twice a day
			*de novo* HBV		Micophenolate mofetil
		Donor		(-)	
	2	Recipient		Mild interface hepatitis	Tacrolimus: twice a day
					Micophenolate mofetil
					Steroid
		Donor		(-)	
	3	Recipient			Tacrolimus: twice a day
			HV stenosis	Marked bridging fibrosis	Micophenolate mofetil
					Steroid
		Donor		(-)	
	4	Recipient	Unstable liver function	Mild lobular hepatitis	Cyclosporine: twice a day
					Micophenolate mofetil
Comparable NTCR Group		Donor		Moderate steatosis	
	5	Recipient	*de novo* HBV	(-)	Cyclosporine: twice a day
			(seroconversion)		Micophenolate mofetil
		Donor			
	6	Recipient	Liver dysfunction	Marked bridging fibrosis	Tacrolimus: twice a day
			HV stenosis		Micophenolate mofetil
					Steroid
	7	Donor		(-)	
		Recipient	None	Mild interface hepatitis	Tacrolimus: twice a day
		Donor		Moderate steatosis	
	8			(NASH suspected)	
		Recipient	Biliary stricture	Canalicular cholestasis	Cyclosporine: twice a day
		Donor		(-)	
	9	Recipient	HV stenosis	Mild interface hepatitis	Cyclosporine: twice a day
					Micophenolate mofetil
	10	Donor		(-)	
		Recipient	None	(-)	Complete cessation
	11	Donor		(-)	
		Recipient	None	(-)	Tacrolimus: once a week
		Donor		Moderate steatosis	
	12	Recipient	Unstable liver function	(-)	Tacrolimus: twice a day
					Micophenolate mofetil

HBV: hepatitis B virus, HV: hepatic vein, NTCR: normalized telomere centromere ratio, NASH: none-alcoholic steatotic hepatitis, (-): almost normal.

Lower NTCR group: Median NTCRs of the recipient hepatocyte was significantly lower than those of the donor.

Compatible NTCR group: Median NTCRs of the recipient hepatocyte was not significantly lower nor higher than those of the donor.

Episodes of acute cellular rejection (ACR) are summarized in Table S3. Liver biopsy was indicated when any liver function parameters (aspartate aminotransferase, alanine aminotransferase, gamma-glutamyl transpeptidase, and total bilirubin) showed abnormal values suggesting ACR. All episodes of ACR were diagnosed on the basis of liver biopsy pathology. Highly experienced pathologists evaluated the degree of portal infiltration by lymphocytes, bile duct inflammation or damage, and venous endothelial inflammation, according to the Banff scheme.

### Preparation of biopsy specimens and histological assessment

The liver specimens were obtained by ultrasonography-guided core needle (16 gauge) biopsy from the donor and recipient under local anesthesia. These paired biopsies were performed within a week of each other. The donors fully approved biopsy of their liver because they expressed a desire to know the pathological state of the liver and the pathological relationship between the donor and recipient livers after receiving a full explanation about all the potential complications of the biopsy.

The liver biopsy specimens were fixed in 10% buffered formalin solution and embedded in paraffin. The specimens were then sliced into sections 3 μm thick for hematoxylin and eosin (HE) staining and into sections 2 μm thick for Q-FISH. All sections included more than 6 portal tracts. Histological findings were assessed by a histopathologist who was an expert in liver pathology (K.M), and described based on the criteria proposed by the Banff Working Group [30].

### Telomere length quantification by Q-FISH

The slides were processed with the Q-FISH method, as reported previously [18], [26]. In brief, the telomeres were labeled with Cy3-labeled CCCTAACCCTAACCCTAA peptide nucleic acid probe (teloC: F1002; Fasmac, Japan) and the centromeres were labeled with a FITC-labeled CTTCGTTGGAAACGGGGT peptide nucleic acid probe (CENP1: a non-specific centromere probe) (custom-made, Fasmac). The chromosome preparations were counterstained with 40, 6-diamino-2-phenylindole (DAPI, Molecular Probes, Eugene, OR, USA). Microscopical images were captured with the Image-Pro Plus software package (version 5.0, Media Cybernetics Co. Ltd., Silver Spring, MD, USA), and analyzed using our own telomere analysis software ‘TissueTelo Ver. 2’. The telomere length estimate for each nucleus was defined as the ratio of the detected signal intensity of the telomere relative to that of the centromere (telomere-to-centromere ratio: TCR). At least 127 cells (median: 208 cells, range: 127–463 cells) were analyzed in each case.

### TCR normalization using a cell block

As a control for variations in sample preparation, we also performed Q-FISH on a cell block-section of a cultured fibroblast strain, TIG-1 [31] (34 population doubling levels, terminal restriction fragment length determined as 8.6 kilo base pairs), placed on the same slide as each hepatic section. Every TCR for hepatocytes was divided by the median TCR for the cell block on the same slide to give the normalized TCR (NTCR) [26].

### Limitation of the study

The main limitation of this study was the small number of subjects for whom NTCR values for the paired donor and graft could be determined. However, despite the limited number (12 pairs) of informative cases, the major findings were statistically significant.

### Statistical analyses

The values of measured variables were expressed as mean ± standard deviation or median with a range of values. The median NTCRs of the donor and recipient hepatocytes were compared between cases using the Mann-Whitney test or Wilcoxon test. Correlations were analyzed with the Spearman correlation coefficient test and a single regression analysis using the software package Dr.SPSS II (SPSS, Chicago, IL). The relationships among NTCR values, donor age and group (lower versus comparable NTCR) were assessed by multiple regression analyses using StatView software version 5.0 (SAS Institute Inc.). Differences were considered significant at *p*<0.05.

## Results

### Quantification of telomere length in liver tissue of donors and recipients using Q-FISH

**Figure 1:**
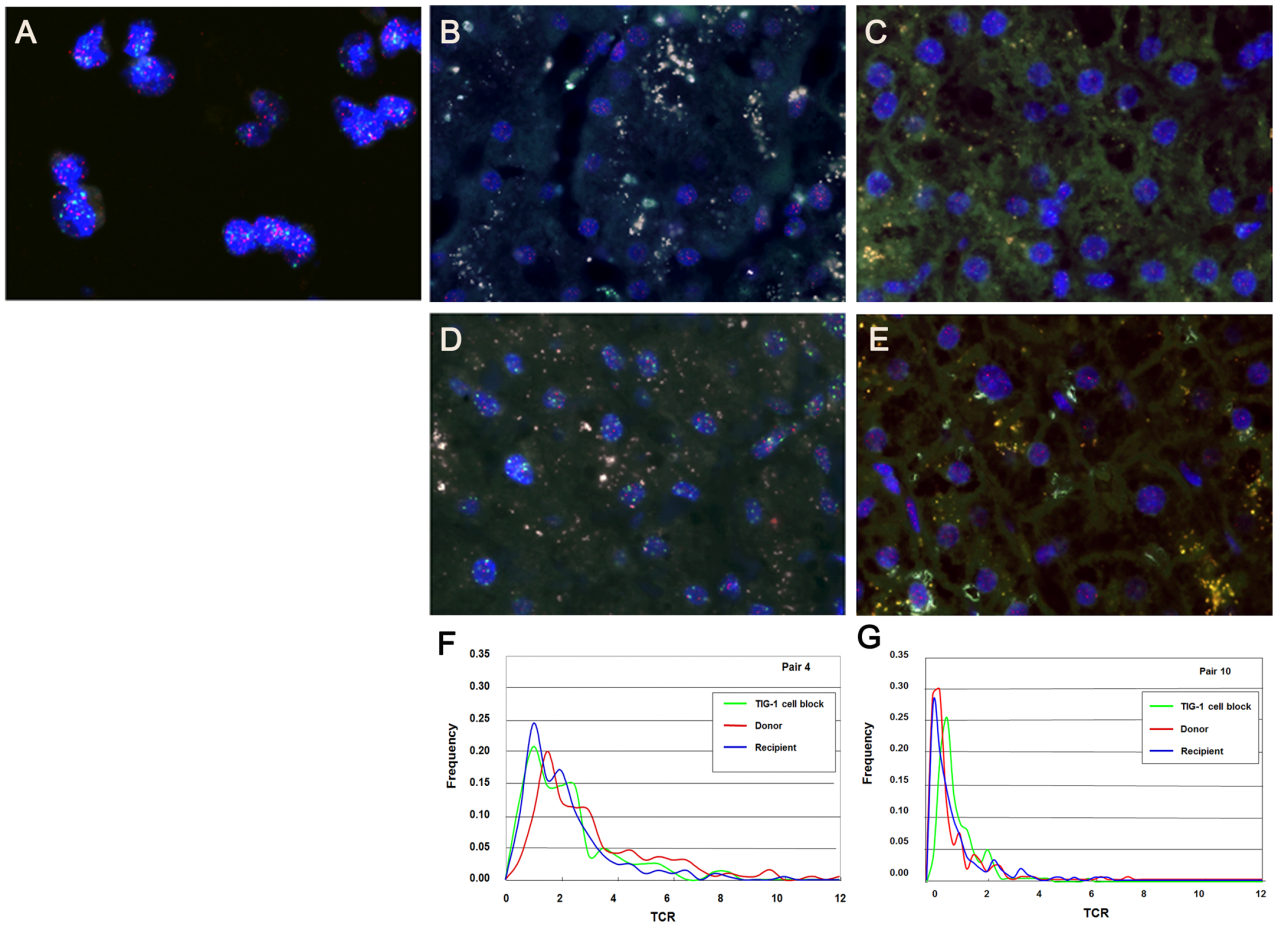
Representative Q-FISH images of TIG-1 and paired liver tissues (red: Cy3, telomere signals; green: FITC, centromere signals; blue: DAPI, nuclei). (A) Q-FISH image reveals the TIG-1 cell block placed on the same slide together with liver sections. Telomere (Cy3, red) and centromere (FITC, green) signals are evident (original magnification ×400). (B) Q-FISH images of pair 4 donor. Telomere and centromere signals are evident in the nuclei (original magnification ×400). (C) Q-FISH images of pair 10 donor. (original magnification ×400). (D) Q-FISH image of pair 4 recipient in the lower NTCR group reveals weaker telomere signals (red) than those in the paired donor (Figure 1B) (original magnification ×400). (E) Q-FISH images of pair 10 recipient, showing brighter telomere signals (red) than those in the paired donor (Figure 2D) (original magnification ×400). (F) Distributions of the telomere intensity given by telomere-to-centromere ratio (TCR) in TIG-1 and hepatocytes from paired liver tissues of pair 4 samples. Green: TIG-1 cells in a cell block, red: hepatocytes in donor, blue: hepatocytes in recipient grafted liver. (G) TCR distribution in TIG-1, and hepatocytes from paired liver tissues of pair 10 samples.

**Figure 2:**
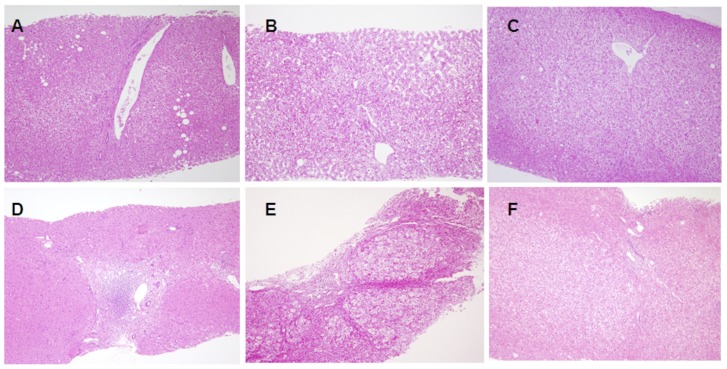
Representative histological and histopathological features of the livers. (A) Histological findings in pair 1 donor, showing almost normal feature (HE, original magnification ×100). (B) Histological findings in pair 3 donor, showing almost normal feature (HE, original magnification ×100). (C) Histological findings in pair 10 donor, showing almost normal feature (HE, original magnification ×100). (D) Histological findings in pair 1 recipient in the lower NTCR group, showing mild interface hepatitis due to infection with hepatitis B virus via the graft from the HBV carrier donor (HE, original magnification ×40). (E) Histological findings in pair 3 recipient in the lower NTCR group, showing marked bridging fibrosis due to repeated hepatic vein stenosis (HE, original magnification ×40). (F) Histological findings in pair 10 recipient, with good liver function and complete withdrawal of immunosuppressant, showing almost normal feature (HE, original magnification ×100).

Figure 1A shows a Q-FISH image of a TIG-1 cell block placed on the same slide as liver sections from the donor and recipient. Representative Q-FISH images of liver tissues from the donor and recipient are shown in Figure 1B, D (pair 4), and Figure 1C, E (pair 10), in which the telomere signals (Cy3) appear red while the centromere signals (FITC) appear green on the DAPI-stained nuclei. With reference to HE-stained serial sections (Shown in Figure 2), we were able to distinguish hepatocytes from other cell types (including ductal cells, interstitial fibroblasts and infiltrating lymphocytes). The profiles of the measured telomere:centromere ratio (TCR) are plotted in Figure 1F, G. Since the frequency of TCR values did not show a normal distribution, we chose the median values as representative. The median values of the TCR for the donor and recipient hepatocytes are divided by the TCR value for TIG-1 cells and obtained normalized TCR (NTCR) (summarized in Table 1). Among interstitial cells, strong signal intensity for Cy3 was often evident in fibroblasts.

### Relationship between NTCR values for the donor and recipient

**Figure 3:**
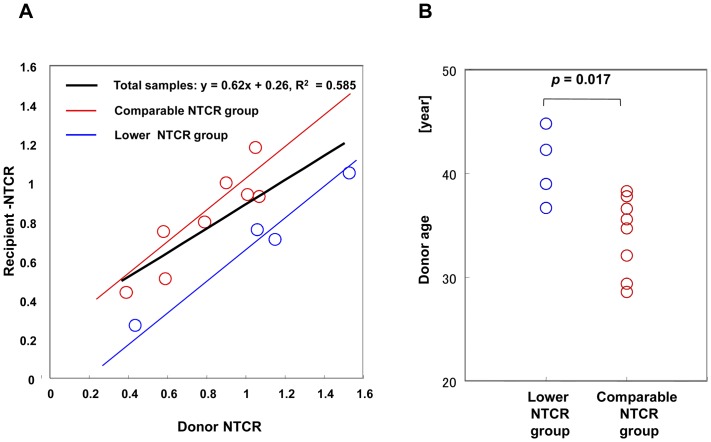
(**A**) Scatter plot analysis of the relationship between NTCRs in donor hepatocytes and those in recipient hepatocytes. A regression line was obtained from all of the paired subjects (n = 12), y (recipient NTCR) = 0.62x(donor NTCR)+0.26, (*r* = 0.765, R^2^ = 0.544, *p* = 0.0038), shown by a solid black line. By using model 1: Y = β_0_+β_1_X_1_ (age)+β_2_ X_2_ (group), that related Y (recipient NTCR) to X_1_ (donor NTCR) and X_2_ (group), given that the value for the comparable group was 1 and that for the lower group was 0. This model yielded a regression line from the comparable patient group (n = 8): Y (recipient NTCR) = −0.10+0.79X(donor NTCR) +0.32, shown by a red line; and a regression line from the lower patient group (n = 4): Y (recipient NTCR) = −0.10+0.79X (donor NTCR), shown by a blue line. The difference of recipient NTCR values between the groups was 0.32 and significant (*P* = 0.001). Red circles: the recipient-donor pair categorized as the comparable NTCR group. Blue circles: the recipient-donor pair categorized as the lower NTCR group. (**B**) Comparison between donors in the comparable group and those in the lower group by age factor. The difference of donor ages (median values) between the groups was 5 y 6 m and significant (*p* = 0.017). Red circles, the donors categorized as the comparable NTCR group. Blue circles, the donors categorized as the lower NTCR group.

Scatter plot analysis of the median NTCR values for hepatocytes provided a robust correlation between donors and recipients (*p* = 0.0038) (Figure 3A). The slope provided by the regression analysis was less than 1, and the mean of the median NTCR value for hepatocytes from donors and that of hepatocytes from recipients (grafts) was 0.82 and 0.78, respectively. Hence, the NTCR values for recipients generally tended to be lower than those for the donors. However, in individual cases, even if the NTCR value for the grafted liver was higher or lower than that for the donor liver, the difference was not statistically significant (*p* = 0.092).

**Figure 4:**
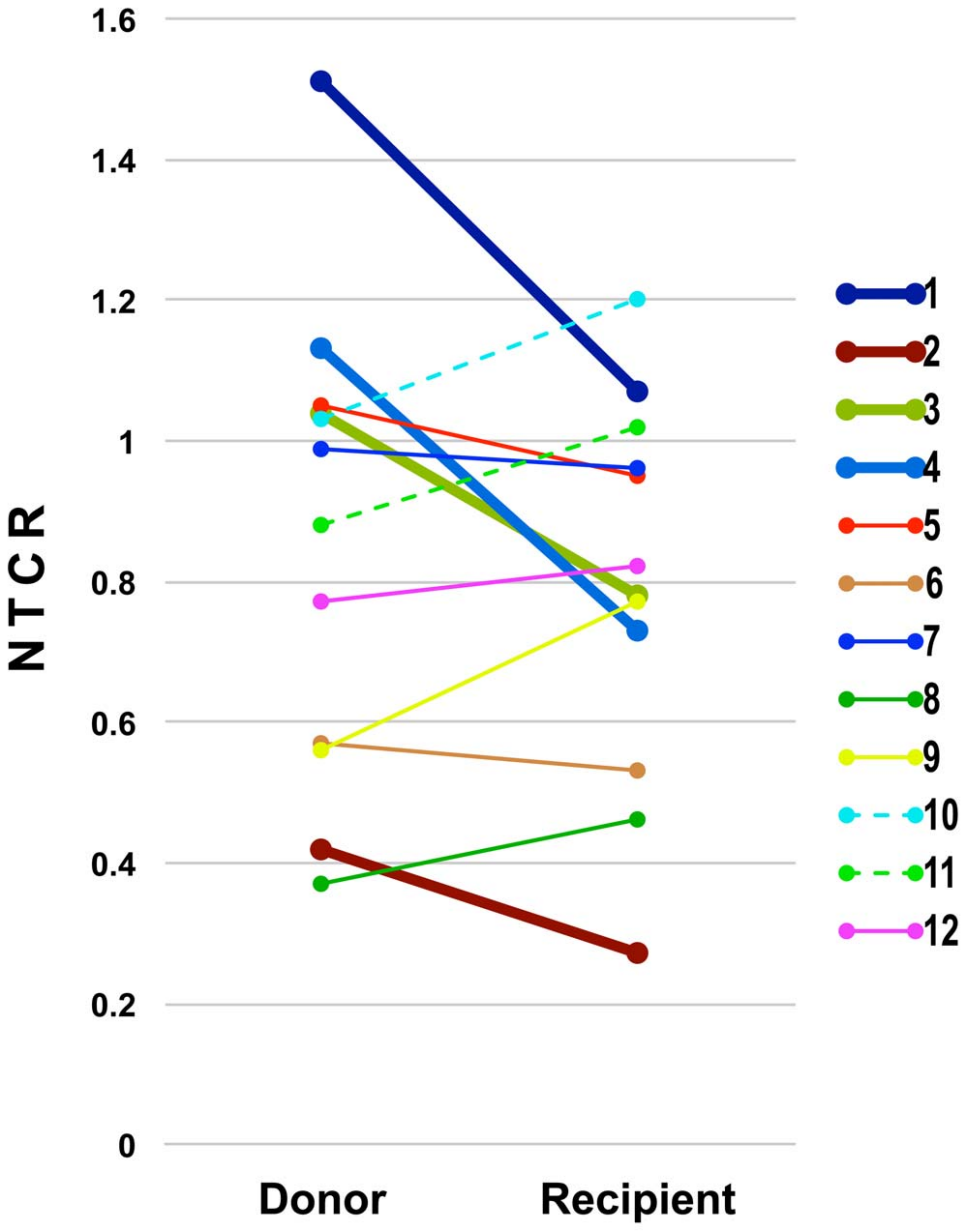
Comparison between donor and recipient NTCR values. Bold lines connect lower NTCR group pairs, and thin lines connect comparable NTCR group pairs. Dashed lines connect the pairs (10 and 11), who achieved complete withdrawal of immunosuppression.

From the relationship between the hepatocyte NTCR values for the donor liver and the graft, we found 4 sample pairs in which the NTCR values for the graft hepatocytes were significantly lower than those for remaining hepatocytes in the donor (Table 1, Figure 4). In the other 8 pairs, although the NTCR values for graft hepatocytes were higher than, comparable to, or lower than the corresponding values for the hepatocytes remaining in the donor, the differences were not statistically significant (Table 1, Figure 4). Hence, we categorized the former as a lower NTCR group and the latter as a comparable NTCR group. Intriguingly, the donors were significantly older in the lower NTCR group (median age 40 y 8 m) than in the corresponding comparable NTCR group (median age 35 y 2 m) (*p* = 0.017) (Figure 3B). Conversely, the oldest one-third of the donors (pairs 2, 4, 3, 7) demonstrated decreased NTCR values in the recipient, and the youngest one-third of the donors (pairs 9, 12, 10, 11) showed increased NTCR values in the recipient.

Next, we applied a multiple regression model to assess the difference between the two groups (see Figure 3A legend). This model yielded a regression line indicating that the average ratio of the recipient NTCR to the donor NTCR was 0.794 (*p*<0.0001), and that the difference between the groups was significant (*p* = 0.001) (Figure 3A).

With regard to other factors, the ages of the recipients at LDLT showed no significant difference between the two groups (lower NTCR group: median age 1 y 10 m, comparable NTCR group: median age 2 y 1 m, *p* = 0.799). Also, neither the NTCR values for recipients nor those for the donors differed significantly between the two groups (0.73 vs. 0.89, *p* = 0.570; 1.04 vs. 0.83, *p* = 0.241, respectively. The period between LDLT and liver biopsy showed no significant differences between the two groups (lower NTCR group: median duration 8 y 5 m, comparable NTCR group: median period 9 y 8 m, *p* = 0.734).

### Histopathological findings in donors and recipients

The liver biopsy samples from all 4 donors in the lower NTCR group showed normal histological findings. On the other hand, three (pairs 5, 8, and 12) of the 8 donors in the comparable NTCR group showed moderate steatosis.

Among the liver biopsy samples from recipients, significant abnormalities were observed in all of the 4 recipients in the lower NTCR group (mild interface hepatitis in cases 1 and 2, mild lobular hepatitis in case 4, marked bridging fibrosis in case 3) and in 4 of the 8 recipients in the comparable NTCR group (marked bridging fibrosis in case 6, mild interface hepatitis in cases 7 and 9, canalicular cholestasis in case 8) (Figure 2, and summarized in Table 2).

### Immunological findings in grafts

In the comparable NTCR group, 3 recipients (pairs 10, 11 and 12) showed a benign course after LDLT, and were treatable with a calcineurin inhibitor at a frequency of less than once a day, or without any immunosuppressant. In particular, the recipient in pair 10, who achieved complete withdrawal of immunosuppression, had a higher median NTCR than the donor, but the difference was of borderline statistical significance (*p* = 0.056). The recipient in pair 11, who has recently achieved complete withdrawal of immunosuppression, also had a higher median NTCR than the donor. In only one recipient (pair 8) who received a graft from a non-father non-mother (stepfather) donor and was treated with a calcineurin inhibitor, the median NTCR was also higher than that of the donor, but the difference was of borderline statistical significance (*p* = 0.065). While only one of 8 recipients in the comparable NTCR group (pair 6) continued to require oral maintenance steroid therapy, 2 of 4 recipients (pairs 2 and 3) in the lower NTCR group did so.

Episodes of acute cellular rejection (ACR) were observed in 2 cases in the lower NTCR group and 4 cases in the comparable NTCR group. Among the cases associated with ACR, 4 (pairs 2, 3, 5, 6; cases 2 and 3 being statistically significant) showed decreasing NTCR values in the recipients, and 2 (pairs 8, 12) showed increasing NTCR values in the recipients, but not to a significant degree. With regard to steatosis, 3 of the 4 cases in the comparable NTCR group associated with ACR in the recipient showed steatosis in the donor.

### Laboratory data for donors and recipients

The laboratory data for recipients (summarized in Table S1) at liver biopsy have so far revealed no significant differences between the lower NTCR group and the comparable NTCR group. In addition, the PELD scores for recipients (summarized in Table S2) have shown no significant differences between the two groups.

## Discussion

In parallel with the increasing need for LDLT, the issue of organ shortage continues to extend worldwide and has become a serious problem. Therefore, the use of organs from marginal donors, such as the elderly, has been increasing [2]. Currently, LDLT is able to ensure survival for more than 5 years, mainly because of improvements in immunosuppression regimens [4], [5], [6]. However, there is still insufficient knowledge of factors that would facilitate long-term or life-long viability or function of the grafted liver after LDLT. Accumulated studies have demonstrated that higher donor age is a risk factor for early relaparotomy and poor outcome [32], [33]; however, some reports have suggested that liver grafts from elderly donors can yield a favorable outcome [34], [35]. Thus, the question of whether the use of grafts from elderly donors may influence the long-term outcome of the graft after LT/LDLT remains unsettled. In addressing possible factors that might have a crucial impact on long-term prognosis, we speculated that hepatocyte telomere length in the recipient might be an indicator of long-term survival potential, as telomere length reportedly reflects not only cellular senescence but also possibly organ aging [36], [37], [13], [38].

The present study represents the first attempt to provide statistical support of telomere length differences in hepatocytes obtained by liver biopsy at almost the same time from both donors and recipients followed up for more than 5 years after pediatric LDLT using the Q-FISH method. The main findings were as follows: Firstly, based on the relationship between the NTCR of the recipient and that of the donor, recipients were classifiable into two groups: a comparable group (showing no significant difference in NTCR) and a lower group (showing a significantly lower NTCR in the recipient than in the donor). However, in 5 of the 8 cases in the comparable group, hepatocyte NTCR values for recipients were larger than those for the donors. Secondly, donors in the lower NTCR group were significantly older than those in the comparable NTCR group, and there was no relationship between the absolute NTCR values *per se* in the two groups, nor with any of the recipient conditions examined.

With regard to the methodology used for telomere length measurement, numerous approaches have been adopted, including Southern blotting, PCR-based methods, and Q-FISH. Previous studies using Southern blotting have shown that fibrosis or hepatitis can accelerate telomere shortening in the liver [15], [39], [40] in recipients with immune-mediated injury and/or pathological abnormalities of the liver, where the telomere length might be shorter than that of donors [41]. This approach has an underlying problem in that hepatic tissue includes a variety of cell types, such as hepatocytes, macrophages, and fibroblasts, whose telomere lengths are measured simultaneously. On the other hand, Q-FISH makes it possible to analyze the length of hepatocellular telomeres in a cell type-specific manner, although precise calibration is required. We have measured telomere lengths in various human organs and tissues by the Q-FISH method utilizing the centromere signal as an internal control and the terminal restriction fragment length defined fibroblasts as an external control [26], [18], [42]. Recently, we have demonstrated telomere length shortening in the hepatocytes of biliary atresia with severe inflammatory changes and fibrosis using the present Q-FISH method, but failed to reproduce our findings by authentic Southern blotting [43]. The robust correlation between the paired values found in the present study further demonstrated that our Q-FISH method was reliable for cell type-specific analysis.

Our previous population studies demonstrated that telomeres in the liver shorten most rapidly with age among those in the major organs. The telomere shortening in liver is especially rapid in infants, and then the rate of shortening slows from adolescence to middle age; no significant decrease is evident from forties to centenarian age [12], [13], [38]. Hence, from the viewpoint of telomere dynamics, the most critical period for liver aging is assumed to be from birth to forties. Our present data, based on a donor population ranging in age from 28 y 7 m to 44 y 10 m, indicated that donors in the lower NTCR group were significantly older than those in the comparable NTCR group. Taken together, the data strongly suggest that the mechanisms for maintenance of telomere length in the grafts were affected by aging-related changes during early adulthood.

The regeneration of a liver graft in a recipient suggests that the structure and function of the graft might be largely controlled by the host environment [44], organ chimerism, or cell migration from the host to the transplanted liver [45], and that telomerase may play a pivotal role in maintaining telomere length and chromosomal stability in proliferating cells [46]. In relation to LT/LDLT and telomere shortening, several candidate causative factors have been examined, including chronic inflammation, fibrosis, and steatosis. Nakajima *et al*. have reported that non-alcoholic fatty liver disease with steatosis causes prominent telomere shortening in hepatocytes [47], and therefore steatosis in a grafted liver might potentially accelerate telomere shortening. In the present study, 3 of 12 donors had moderate steatosis (pairs 5, 8, and 12), and all were categorized in the lower NTCR group. Hence, these cases would require careful attention. Liver fibrosis is another histopathological feature of liver disease, and has been reported to be a significant indicator of prognosis after LDLT [48]. In the present study, 2 of the 12 recipients showed marked fibrosis (pairs 3 and 6); both were treated with continuous steroid, but were separated into different groups. Hence, to date, we have been unable to confirm the relationship between fibrosis and telomere length. Hepatitis after LT/LDLT is another critical complication. Three of the lower NTCR group (cases 1, 2, 4) and two of the comparable NTCR group (cases 7, 9) suffered mild hepatitis, and all of them showed a decrease of hepatocyte NTCR in the recipient. These findings are comparable to previous studies [15], [39], [40], [41], and suggest that livers from younger donors may have higher resistance to hepatitis.

With regard to immunological findings, we recently assessed the association of ACR with several factors in 114 LDLTs performed at our institute, including the cases analyzed in the present study. We found that paternal grafts with gender mismatch were associated with a higher incidence of ACR than maternal grafts with gender match, and that there was no significant difference between the donor age groups [49]. Since all of the donors except one in the present study were male, it was not possible to examine any influence of donor gender. However, the latter conclusion is compatible with our present findings. Intriguingly, some graft hepatocytes, including those in pairs 10 and 11, where complete withdrawal of immunosuppression was possible, showed a larger NTCR than those of the donor. Although the difference was not statistically significant, our findings strongly suggest that the telomeres in grafted livers might be elongated somewhat longer when the grafts are immunologically well controlled. Human leukocyte antigen (HLA) compatibility matching is indisputably important in kidney, heart, and bone marrow transplantation, but is not largely considered to have a clinically significant impact in liver transplantation [50], [51]. In the present study, we were unable to demonstrate any significant effect of HLA compatibility on telomere dynamics.

With regard to the relationship between recipient status and LT/LDLT outcome, some studies have demonstrated a correlation between the PELD score before LT and outcome after LT [6], [21]. However, we were unable to find any significant relationship between NTCR dynamics and host status, including PELD score, in this series.

Major arguments concerning the clinical impact of donor age on LT/LDTP outcome have focused mainly on the elderly (60∼65 years of age or more). However, aging phenomena have been observed not only in the elderly but also in young adult or middle-aged individuals [33]. For example, concentrations of growth-hormone/insulin-like growth factor reportedly begin to decline from early adulthood (∼20 years of age), and muscle area/strength from 30 years of age [52], [53]. Our previous population studies of telomere length have demonstrate that telomeres in the skin start to shorten at around 30 years of age [54]; on the other hand, those in the liver shorten rapidly during young age, and continue to do so at 30 to 40 years of age, and then reach a stationary state [12], [13]. The present data provide further evidence for the aging process that occurs during youth and middle age, and shed more light on the importance of the aging process during this earlier period before old age.

Notwithstanding our retrospective study design and the limited number of samples analyzed, our findings lend additional support to the validity of LDLT, because telomere lengths in the majority of the recipients were statistically comparable to those in the native liver of the donor. Furthermore, telomere lengths showed a tendency to be elongated in well-controlled grafted livers. Our data also provide essential clues to the relationship between hepatocyte aging and LDLT outcome, particularly with regard to young/middle-aged individuals. Further studies will undoubtedly reveal other factors that affect hepatocyte telomere length in the grafted liver and their long-term effects on the graft after pediatric LDLT.

## Supporting Information

Table S1 **Laboratory data of recipients and donors at biopsy.** Lower NTCR group: Median NTCRs of the recipient hepatocyte was significantly lower than those of the donor. Comparable NTCR group: Median NTCRs of the recipient hepatocyte was neither significantly lower nor higher than those of the donor. AST: aspartate aminotransferase, ALT: serum alanine aminotransferase, γ-GTP: γ-glutamyl transpeptidase, Alb: albumin, T-bil: total bilirubin, PT-INR: international normalized ratio of prothrombin time.
(DOC)

Table S2 **Pediatric End-stage Liver Disease (PELD) score of patients.** Alb: albumin, T-bil: total bilirubin, PT-INR: international normalized ratio of prothrombin time.
(XLS)

Table S3 **Acute cellular rejection episodes and Immunosuppression status.** ACR: Acute Cellular Rejection.
(XLS)
