# Supplementary material for: Q-FISH Measurement of Hepatocyte Telomere Lengths in Donor Liver and Graft after Pediatric Living-Donor Liver Transplantation: Donor Age Affects Telomere Length Sustainability
Source: PLoS One. 2014 Apr 11;9(4):e93749. doi: 10.1371/journal.pone.0093749 (PMC3984102; doi:10.1371/journal.pone.0093749)
Supplement: Table S1 — Laboratory data of recipients and donors at biopsy. Lower NTCR group: Median NTCRs of the recipient hepatocyte was significantly lower than those of the donor. Comparable NTCR group: Median NTCRs of the recipient hepatocyte was neither significantly lower nor higher than those of the donor. AST: aspartate aminotransferase, ALT: serum alanine aminotransferase, γ-GTP: γ-glutamyl transpeptidase, Alb: albumin, T-bil: total bilirubin, PT-INR: international normalized ratio of prothrombin time. (DOC) [file pone.0093749.s001.doc]

**Table S1. Laboratory data of recipients and donors at biopsy**

Group Pair No. AST ALT Alb γ-GTP T-bil PT-INR

1 Donor 18 13 4.0 26.0 0.72 0.95

Lower NTCR Group

Recipient 36 39 4.0 12.0 0.57 1.07

2 Donor 25 30 4.4 30.0 0.59 1.01

Recipient 21 19 4.1 17.0 0.99 1.20

3 Donor 19 32 3.9 95.0 0.68 1.12

Recipient 32 23 3.4 36.0 1.03 1.27

4 Donor 15 14 4.1 10.0 0.61 1.01

Recipient 37 22 4.5 24.0 0.79 1.06

Average Donor 19.7 ± 5.0 25.3 ± 9.9 4.1 ± 0.3 45.0 ±44.4 0.63 ± 0.0 1.0 ± 0.1

Recipient 31.5 ± 7.3 25.8 ± 9.0 4.0 ± 0.5 22.3 ± 10.4 0.85 ± 0.2 1.2 ± 0.1

5 Donor 21 30 3.8 28.0 1.11 0.97

Recipient 26 16 3.9 18.0 1.02 1.03

6 Donor 14 11 4.1 16.0 0.78 1.03

Recipient 62 142 3.0 112.0 3.79 1.67

Comparable NTCR Group

7 Donor 29 24 4.3 33.0 0.81 0.94

Recipient 36 40 4.3 37.0 1.09 1.08

8 Donor 25 39 4.4 100.0 1.27 1.09

Recipient 75 108 3.5 199.0 3.14 1.06

9 Donor 14 8 4.2 11.0 0.73 1.05

Recipient 25 19 4.2 45.0 0.38 1.07

10 Donor 26 38 4.2 50.0 0.86 1.00

Recipient 21 14 4.3 16.0 0.51 1.04

11 Donor 23 18 4.0 23.0 0.84 1.05

Recipient 30 13 4.2 16.0 0.73 1.08

12 Donor 24 39 4.3 35.0 0.74 0.95

Recipient 32 15 4.1 18.0 0.81 1.07

Average Donor 22.0 ± 5.5 25.9 ± 12.6 4.2 ± 0.2 37.0 ±28.2 0.89 ± 0.2 1.0 ± 0.1

Recipient 38.4 ± 19.5 45.9 ± 50.4 3.9 ± 0.5 57.6 ± 65.6 1.43 ± 1.3 1.1 ± 0.2

Table S1. Footnotes:

Lower NTCR group: Median NTCRs of the recipient hepatocyte was significantly lower than those of the donor. Comparable NTCR group: Median NTCRs of the recipient hepatocyte was neither significantly lower nor higher than those of the donor.

AST: aspartate aminotransferase, ALT: serum alanine aminotransferase, γ-GTP: γ-glutamyl transpeptidase, Alb: albumin, T-bil: total bilirubin, PT-INR: international normalized ratio of prothrombin time.
